# Phenotypic and Molecular Characterization of *β*-Lactamases among Enterobacterial Uropathogens in Southeastern Nigeria

**DOI:** 10.1155/2020/5843904

**Published:** 2020-02-24

**Authors:** M. C. Ugwu, M. Shariff, C. M. Nnajide, K Beri, U. M. Okezie, I. R. Iroha, C. O. Esimone

**Affiliations:** ^1^Department of Pharmaceutical Microbiology &Biotechnology, Nnamdi Azikiwe University Awka, Anambra, Nigeria; ^2^Department of Microbiology, Vallabhbhai Patel Chest Institute, University of Delhi, Delhi, India; ^3^Department of Applied Microbiology, Ebonyi State University, Abakiliki, Nigeria

## Abstract

Little is known about the molecular basis of antibiotic resistance among uropathogens in Southeast Nigeria. The aim of the study was to characterize enterobacterial uropathogens with respect to drug resistance. One hundred (100) enterobacterial uropathogens were studied. Their antibiotic susceptibility patterns were evaluated using disk diffusion, screened, and confirmed phenotypically for the presence of *β*-lactamases: ESBL, AmpC, carbapenemase, and MBLs. Screen positives were further tested for various *β*-lactamase genes by PCR. Our isolates showed variable resistance to most drugs tested. Out of the 58 ESBL screen positive *E. coli,* 35 were confirmed positive with PCR. The predominant ESBL gene was bla_TEM_ while bla_SPM_ was the most prevalent among MBL genes. Forty-six percentage of the screen positive *Salmonella* isolates coharbored bla_TEM + SHV_ genes. Nine of the 10 ESBL screen positive *K. pneumoniae* were phenotypically and PCR positive. Three isolates of *K. pneumoniae* were positive for MBL genes. All the 10 *C. freundii* were positive for ESBL genes. The study showed high prevalence of drug-resistant genes among the enterobacterial uropathogens. Majority of the uropathogens harbored >1 antibiotic-resistant gene, and the most predominant gene was ESBL (bla_TEM_) followed by the MBL (SPM) gene.

## 1. Introduction

Urinary tract infections (UTIs) are among the commonest human bacterial infections occurring both in the community and hospital settings, particularly in developing countries with a high rate of casualty and financial cost [1, 2]. UTI exist when the number of microorganisms (≥10^5^ cells per milliliter) of urine is detected in properly collected mid-stream clean catch urine [3]. UTIs are caused by a variety of pathogens but mostly by the *Enterobacteriaceae* [1, 4, 5]. Most of the uropathogenic bacteria are from the host bowel flora which enters the bladder through the urethra/bowel reservoir [6, 7]. There have been increasing cases of antibiotic resistance among urinary tract pathogens. Though UTI is treatable, it is now becoming increasingly difficult to control because of antibiotic resistance, especially in the *Enterobacteriaceae* family [8]. As a result, these bacterial uropathogens are of public health concern with huge social and economic challenges [1, 8, 9]. The most common mechanism of resistance among the *Enterobacteriaceae* is the production of hydrolytic enzymes, the “*β*-lactamases” [10]. Complications in UTIs are on the increase because of the increasing prevalence of *β*-lactamases producing uropathogens [4]. Gram-negative bacteria that produce *β*-lactamases are a major concern in healthcare due to their ability to spread globally and the consequent limited treatment options due to the multiple resistance genes as well as the enzymes' associated link with resistance to other non-beta-lactam antibiotics [11–13]. Accurate identification of the antimicrobial resistance of a pathogen is decisive for improved diagnosis, judicious antibiotic use, infection control, and epidemiological surveillance [13].

Molecular genotyping has been used along with phenotyping techniques to screen and confirm expression of antimicrobial drug resistance within a population [11]. Till date, little is known about the molecular basis of antimicrobial resistance in bacteria isolated from UTI in Southeastern Nigeria as inadequate attention has been given to the understanding of the molecular epidemiology of uropathogens in Nigeria, a high-burden country. In appreciation of the above-outlined issues, this study was designed to investigate the antimicrobial susceptibilities, prevalence of *β*-lactamase phenotypes and genotypes among the enterobacterial uropathogens in Southeastern Nigeria.

## 2. Materials Methods

### 2.1. Isolation and Identification

Clean-catch urine samples were collected from patients (who had UTI as their primary diagnosis) attending Anambra State University Teaching Hospital, Amaku, Awka. The isolates were collected between June 2016 and Feb 2017. Verbal informed consent was obtained from all patients prior to specimen collection, and the study was conducted after obtaining due ethical approval from the Anambra State Ministry of Health (MH/COMM/523/68) and the ethical committee of the hospital (COOUTH/AA/VOOL.1.002). No duplicate samples were collected. The bacterial isolates were identified with respect to their cultural and biochemical characteristics.

### 2.2. Antibiotic Susceptibility Study

Antibiotic susceptibility testing was done using Kirby-Bauer's disk diffusion method. The antibiotic disc (Himedia labs, India) containing the following antibiotics was used: cefoxitin (30 *μ*g), ceftazidime (30 *μ*g), cefotaxime (30 *μ*g), cefpodoxime (30 *μ*g), aztreonam (30 *μ*g), meropenem (10 *μ*g), ciprofloxacin (30 *μ*g), ofloxacin (5 *μ*g), norfloxacin (10 *μ*g), levofloxacin (5 *μ*g), cotrimoxazole (25 *μ*g), amoxicillin (10 *μ*g), and gentamicin (10 *μ*g). The inhibition zone diameters (IZDs) produced by the antibiotics were recorded and interpreted as per CLSI guidelines [14].

### 2.3. Screening for ESBL, MBL, Carbapenemase, and AmpC Production

The isolates were screened for ESBL production by checking their susceptibility against the 30 *μ*g disk each of ceftazidime, cefotaxime, cefpodoxime, and aztreonam. The screen positives were confirmed phenotypically by the modified combined disc on a Mueller-Hinton agar supplemented with 200 *μ*g/ml cloxacillin. An isolate was considered an ESBL producer when the IZD around cefotaxime-clavulanate and/or ceftazidime-clavulanate is ≥5 compared with the IZD around the cefotaxime/ceftazidime disc [15, 16].

Meropenem-resistant isolates were further confirmed for MBL production by the meropenem (MRP)-EDTA combined disc test as described by Behera et al. [17]. An isolate was recorded to be MBL positive if there was a difference of ≥7 mm in IZD between the meropenem + EDTA disc and meropenem disc alone [17]. Similarly, the isolates were equally screened for carbapenemase production by checking their susceptibility to meropenem. An organism was considered to be carbapanamase screen positive if the IZD produced by meropenem is between 16–21 mm. The screen positives were confirmed phenotypically using the modified Hodge test (MHT) according to a previously described method [18]. Briefly, standardized inoculums of *E. coli* ATCC 25922 were inoculated on a Mueller-Hinton agar plate. A 10 *μ*g meropenem disk (Himedia, India) was applied aseptically at the center of the inoculated Mueller-Hinton agar plate, and a suspension of the test isolate was streaked from the edge of the meropenem disk (10 *μ*g) to the edge of the Mueller-Hinton agar plate. After incubation at 37°C for 18–24 hrs, the Mueller-Hinton agar plates were observed for cloverleaf effect at the intersection of the test isolate and the *E. coli* ATCC 25922 organisms, within the inhibition zone of the meropenem disk (10 *μ*g). Isolates that were cefoxitin resistant were also screened for the presence of AmpC *β*-lactamase as previously described by Rynga et al. [19].

### 2.4. Molecular Studies

#### 2.4.1. DNA Extraction

DNA extraction was carried out using HiPurATM Bacterial Genomic DNA purification Kit (HIMEDIA, category no MB505-50PR HiPurATM Bacterial Genomic DNA purification Kit) by following the manufacturer's instructions. The extracted DNA was stored at −20°C and used for various molecular studies.

#### 2.4.2. PCR Reactions

The isolates that were screen positive for ESBLs were subjected to multiplex PCR using specific primers for different families of ESBLs (Table 1):

#### 2.4.3. PCR for ESBL (bla_TEM_, bla_SHV_, and bla_OXA-1-LIKE_)

Briefly, multiplex PCR reactions were performed in a final volume of 25 *μ*l of the amplification mixture containing 1.25 U of *Taq* DNA polymerase, 1X *Taq* buffer, 0.2 mM each of dNTPs, 0.2 *μ*M of each primer, and 2 *μ*l of DNA template. The PCR was carried out with a Biorad thermal cycler (UK) using the following conditions: 94°C for10 min; 94°C for 30 sec, 60°C for 40 sec, and 72°C for 1 min for 30 cycles, with a final extension at 72°C for 7 min. PCR products were visualized on a 1.8% agarose gel stained with ethidium bromide.

#### 2.4.4. PCR for ESBL (bla_CTX-M1_, bla_CTX-M2_, and bla_CTX-M9_)

Multiplex PCR reactions were performed in a final volume of 25 *μ*l of the amplification mixture containing 1.25 U of *Taq* DNA polymerase, 1X *Taq* buffer, 0.2 mM each of dNTPs, 0.2 *μ*M of each primer, and 2 *μ*l of DNA template. PCR was carried out with a Biorad thermal cycler (UK) using the following conditions: 94°C for10 min; 94°C for 40 sec, 60°C for 40 sec, and 72°C for 1 min for 30 cycles, with a final extension at 72°C for 7 min. PCR products were visualized on a 1.8% agarose gel stained with ethidium bromide. Similarly, the isolates were further screened for other ESBL genes: bla_VEB_, bla_GES_, and bla_PER_ using specific primers through multiplex PCR.

#### 2.4.5. PCR for MBL, AmpC, and KPC

The 25 isolates that were screen positive for MBLs by the phenotypic test were subjected to multiplex PCR using specific primers for different families of MBLs like bla_VIM_, bla_IMP_, bla_SPM_, bla_SIM_, and bla_GIM_ [19]. The multiplex reaction conditions were 94°C for 5 min; 94°C for 30 sec, 52°C for 40 sec, and 72°C for 50 secs for 36 cycles, with a final extension at 72°C for 5 min. PCR products were visualized on a 1.8% agarose gel stained with ethidium bromide. PCR was equally carried out for AmpC (multiplex PCR) and KPC and NDM (uniplex PCR) using the primers and reaction conditions as in Table 2.

## 3. Results

A total of one hundred (100) enterobacterial uropathogens, *E. coli* (**58**), *Salmonella* (**15**), *K. pneumoniae* (**14**), *Citrobacter freundii* (**10**), and *Enterobacter aerogenes* (**3**), were isolated and identified from 300 urine specimens collected from patients that present with clinical symptoms of UTI and positive urine culture (≥10^5^ CFU/mL).

The antibiotic susceptibility of the isolates shows that most of the *E. coli* isolates (Table 3) were resistant to cefpodoxime, cotrimoxazole, and meropenem, intermediately susceptible to aztreonam, cefotaxime, and ceftazidime but susceptible to the fluoroquinolones. *Salmonella* isolates, on the other hand (Table 4), had a very good susceptibility profile to the 3^rd^ generation cephalosporins (cefpodoxime, ceftriaxione, cefotaxime, and ceftazidime), intermediately susceptible to cefoxitin but were resistant to ofloxacin and cotrimoxazole. *K. pneumoniae* isolates were resistant to cefpodoxime, cefotaxime, and cotrimoxazole but susceptible to the fluoroquinolones (Table 5).  Table 6 shows the summary of multiple antibiotic resistant indices (MARIs) of uropathogens. Only *Salmonella* spp and *E. aerogenes* had a MARI <0.2.

### 3.1. Phenotypic Screening of the Uropathogens for Beta-Lactamase Production

The screening tests showed 96% of the uropathogens (58 *E. coli*, 15 *Salmonella*, 10 *K. pneumoniae*, 10 *C. freundii*, and 3 *E. aerogenes*) were screen positive for ESBL production while 58% (21 *E. coli*, 15 *Salmonella,* 13 *K. pneumoniae*, 6 *C. freundii*, and 3 *E. aerogenes*) were screen positive for AmpC.

### 3.2. Results of Molecular Studies

Out of the 58 ESBL screen positive *E. coli*, 35 (60.3%) were confirmed positive with PCR (Table 7). The predominant gene was bla_TEM_. Forty-two of the *E. coli* isolates were positive for various MBL genes by PCR. bla_SPM_ was the most predominant MBL gene. Ten (10) of the 42 *E. coli* had coexpression of more than one MBL gene: [3(bla_IMP_ + bla_SPM_), **1**(bla_SPM_ + bla_GIM_), **3**(bla_SPM_ + bla_SIM_), 1(bla_SPM_ + bla_VIM_ + bla_SIM_), **2**(bla_IMP_ + bla_SPM_ + bla_GIM_ + bla_SIM_)]. Two out of the 21 AmpC screen positives were phenotypically positive for AmpC and only one of these was confirmed positive by PCR. Only 2 *E. coli* isolates were KPC positive by PCR while none of the *E. coli* isolates was positive for the NDM gene. Seven out of the 15 ESBL screen positive *Salmonella* isolates were confirmed by PCR to coharbor bla_TEM_ + bla_SHV_ genes, 3 isolates harboring bla_CTX-M2_ (*n* = 1), bla_GES_ (*n* = 1) and bla_PER_ gene (*n* = 1). Of the 7 MBL screen positive *Salmonella*, 2 were PCR confirmed positive: 1 (bla_IMP_ + bla_SPM_ + bla_VIM_) and 1 (bla_IMP_ + bla_VIM_ + bla_GIM_). Nine of the 10 ESBL screen positive *K. pneumoniae* were phenotypically and PCR positive, 5 of which had coexpression of bla_TEM_, bla_SHV_, and bla_OXA-1-LIKE_. Of the 13 AmpC screen positive *K. pneumoniae*, none was confirmed to be a AmpC producer. Three isolates of *K. pneumoniae* were positive for MBL genes: bla_IMP_ (*n* = 1), bla_IMP_ + bla_VIM_ + bla_GIM_ (*n* = 1), and bla_IMP_ + bla_GIM_ + bla_VIM_ + bla_SIM_ (*n* = 1). All the 10 *C. freundii* were positive for ESBL genes. Bla_TEM_ was the predominant ESBL gene. It existed in combination with bla_GES_ in 5 isolates and with bla_VEB_ in 1 isolate. Two out of the 21 AmpC screen positives were phenotypically positive for AmpC, and only one of these was confirmed positive by PCR. Only 2 *E. coli* isolates were KPC positive by PCR.

## 4. Discussion

*Enterobacteriaceae* are the highest reported causes of UTI and are usually resistant to several antibiotics resulting in recurrent UTIs, especially in the high-risk population [16, 20, 21].

They present a public health challenge and thus deserve an adequate attention. For an in-depth understanding of the underlying resistance genotypes and/mechanisms, this study characterized the enterobacterial uropathogens with respect to drug resistance and their *β*-lactamase production capacities. Antibiotic resistance is a key clinical and public health challenge in treating UTI. Emergence of *β*-lactamase producers among the *Enterobacteriaceae* reduces therapeutic options because the isolates often coexpress resistance to other classes of antibiotics. Our predominant isolates *(E. coli*, *Salmonella* spp., and *K. pneumoniae)* showed variable resistance to most antibiotics tested. This is similar to the findings of Ekwealor et al. [1]. The fluoroquinolones and gentamicin were highly active against *E. coli* isolates and thus can be prescribed for the empiric treatment of UTI caused by *E. coli.* Similarly, in Libya, Abubaker et al. [5] reported a very good susceptibility of uropathogenic *E. coli* to ciprofloxacin, and a very low resistance to gentamicin was equally reported by Elsayed et al. [4] in Egypt.

Unlike the *E. coli* isolates, the *salmonella* spp. was resistant to the fluoroquinolones. The susceptibility test for *K. pneumoniae* showed that amoxicillin, cefpodoxime, cefotaxime, aztreonam, and cefoxitin exhibited very poor antipneumococcal activity while the fluoroquinolones showed very good activity and is in agreement with the reports of Sikarwar & Batra [22] that a fluoroquinolone, ciprofloxacin, had a 90% antibacterial activity against uropathogens. It was observed that *K. pneumoniae* isolates (Table 5) were more resistant to most of the antimicrobial agents tested than *E. coli* and *Salmonella* isolates. A similar scenario of multidrug resistance (MDR) of uropathogenic *Klebsiella* spp. has been reported in Libya [5]. It should be noted that all the isolates had poor susceptibility to cotrimoxazole and amoxicillin. This is in agreement with what was reported in Ethiopia where a high level of resistance (>70%) was recorded for cotrimoxazole and ampicillin by uropathogens [23]. The observed low susceptibility might be connected with the misuse of the agents as cotrimoxazole and ampicillin were the first choice of drugs for the empirical treatment of UTI [23]. Several researches have reported increasing prevalence of trimethoprim-sulfamethoxazole-resistant uropathogenic strains and suggested fluoroquinolones as an alternative treatment choice for UTI [24]. *E. coli* and *Salmonella* were very sensitive to aztreonam and ceftazidime. This observed low resistance rates may be due to less use of these drugs in treating bacterial infections in Nigeria. A significant sensitivity to gentamicin was noted with *E. coli* and *C. freundii* (Tables 3 & 8). Two related studies in Abakilikii and Enugu both in Southeastern Nigeria equally reported a remarkable susceptibility of uropathogens to gentamicin [18, 25]. This might be because gentamicin being a parenteral preparation might be used with much restriction. Improper antibiotic use, dose, and duration of administration have been reported as predisposing factors for the emergence of antibiotic-resistant strains in a locality [4]. Commonly, in our hospitals ceftriaxione is used empirically for inpatients and amoxicillin-clavulanate for outpatients by the physicians. The choice of drug treatments will further be determined by the sensitivity tests.

Sixteen (27.6%) of the screen positive *E. coli* were phenotypically confirmed to be ESBL producers (Table 9). Similar rates (27.7%) of ESBLs have been reported from a neighboring southeastern state, Enugu, by Ejikeugwu et al. [18] and 26.1% in southwestern Nigeria [26]. Lower prevalence (6.7%) of ESBLs was detected phenotypically among uropathogenic *E. coli* in northwestern Libya [5]. However, higher prevalence of ESBL-producing uropathogenic *E. coli* (38.9%) was reported in Nepal [11], 40% in Potohar region of Pakistan by Ali et al. [24], and 83% in Doha, Qatar [20]. The rates of resistance of ESBL-producing bacteria to antibiotics have previously been reported to be geographically dependent. This is due to the differences in antimicrobial usages and infection control measures in these locations [27].

On the molecular level, the prevalence of ESBL production was *E. coli* (60.34%), *C. freundii* (100%), *K. pneumoniae* (64.28%), and *Salmonella* spp. (46.66%). These high rates are of serious issue as the spread of these enzymes is normally driven by mobile genetic elements which facilitate the horizontal transmission of the resistance genes among bacteria of other species [28]. In addition, they often carry genes that encode high levels of resistance to many other antibiotics and cause high therapeutic failures among infected patients [16, 29]. The increasing prevalence of infections caused by antibiotic-resistant bacteria makes the empirical treatment of UTI difficult and the outcome unpredictable. It is thus associated with higher cost of therapy, increased risk of complications, morbidity, and mortality [4, 16]. Many studies reported that urine of UTI patients harbors ESBL-producing *E. coli* [5, 30]. A similar observation was noted by Iroha et al. [31] in the neighboring Enugu state where 81.8% of ESBL-producing strains of *E. coli* was isolated from urine of outpatients in a tertiary care hospital. ESBLs have been reported among 51–90% of *Enterobacteriaceae* in Asia. Similar to our findings, Padmavathy et al. [32] reported that the percentage of ESBL-producing *E. coli* was 66.9% in Chennai, India.

The high levels of ESBL producers are a major threat to infection management as this may have contributed to the antibiotic resistance reported in this study. ESBL-producing organisms are known to contain plasmids with genes that encode resistance to quinolones, aminoglycosides, and cotrimoxazole. This is exemplified in the resistance profile of *K. pneumoniae* (Table 5). The high prevalence of bla_TEM_ among the *C. freundii* isolates (Table 8) might be responsible for their high resistance to the *β*-lactams {amoxicillin (80%), cefpodoxime (80%), and ceftazidime (60%)} as observed in Table 6. It has been reported previously that resistance to oxyimino-cephalosporins (e.g., cefpodoxime and ceftazidime), is caused mostly by TEM-type of ESBL [14]. However, ESBL-producing *E. coli* and *C. freundii* isolates were susceptible to fluoroquinolones. This finding is in line with a similar study done in Southeastern Nigeria by Iroha et al. [33]. They advised limited use of any cephalosporin on an ESBL positive *E. coli* infection. Since *E. coli* isolates showed high prevalence of resistance to various antibiotics, strategies to control the increase in resistant uropathogens would be important. The observed low resistance of *E. coli* (13.8%) and *Salmonella* spp (13.3%) to cefotaxime and high susceptibility to ceftriaxone (>80%) might be due to the low prevalence of bla_CTX-M_ gene in this study. This analogy can also explain the high resistance profile of *K. pneumoniae* (64.9%) to cefotaxime as 5 of the 14 *K. pneumoniae* isolates harboured the bla_CTX-M1_ gene. Among the Gram-negative pathogens, bla_CTX-M_ genes have been reported as a vital mechanism of resistance to cefotaxime and ceftriaxone [8]. Our findings are in line with the reports of Eskandari-Nasab et al. [34] in which the bla_CTX-M_ genes were predominant in *Klebsiella* spp. Similarly Kuldeep and Nitika [21] stated that majority of ESBLs in *E. coli* are derived from the common plasmid mediated broad-spectrum bla_TEM_. Majority of ESBLs are derived from plasmid mediated penicillinases of the TEM and SHV families [35]. Low levels of bla_GES_, bla_VEB_, and bla_PER_ were reported in this study. It has been stated that the most frequently detected clinically important ESBLs belong to the TEM, SHV, and CTX-M families while GES, VEB, and PER are of less prevalence [28, 36]. Although, the frequency of ESBL-producing isolates is increasing, the rate of infection can be minimized by regular surveillance and monitoring in order to institute effective and credible treatment of UTI.

MBLs have been recognized as one of the most notable resistance determinants in *Enterobacteriaceae* [37]. The SPM gene was the most predominant MBL gene in our study. There was mixed expression of the MBL genes among our isolates. Ten (10) of the 25 MBL screen positive *E. coli* had coexpression of more than one MBL gene. There are increasing reports of MBL-producing Gram-negative bacteria in southeastern Nigeria. Ejikeugwu et al. [38] had reported high occurrence of MBL-producing *E. coli* and *Klebsiella* species from an abattoir. Since the genes that code for MBL production in Gram negatives are chromosomally or plasmid mediated, they can easily be transmitted through mobile genetic elements among bacterial population in a community [39]. The discrepancy in the percentage of phenotypic and genotypic *β*-lactamase confirmed producers (Table 9) might be because of coexpression of more than one ESBL, MBL, and/or AmpC genes in an organism. Occurrence of multiple ESBL types and/or ESBL-AmpC combinations within the same organism has previously been reported to make phenotypic identification of the *β*-lactamases difficult and not reliable [32]. It might also be that the genes detected by PCR are not effectively expressed phenotypically [40]. Similarly, Krishnamurthy et al. [35] observed a significant difference in detection of ESBL positive isolates by phenotypic and genotypic methods. They attributed it to lower sensitivity of the phenotypic method and the influence of environmental factors and maintained that the genotypic method has a 100% specificity and sensitivity as it uses specific PCR amplification of resistance genes.

We confirmed low prevalence of AmpC and KPC genes among our uropathogens while none of the *E. coli* isolates was positive for NDM genes. The AmpC producer was also found to be ESBLs negative. The low prevalence of AmpC genes in our study is likely to be responsible for the observed high susceptibility of *E. coli* (75%) and intermediately susceptible of *Salmonella* to cefoxitin. Conversely, a study in Chennai, India, reported that 61.9% of the uropathogenic *E. coli* isolates expressed an AmpC phenotype [32].

## 5. Conclusion

The uropathogens were found to be resistant to various antimicrobial classes studied. The study showed high prevalence of drug-resistant genes among the enterobacterial uropathogens. Majority of the enterobacterial uropathogens harbored more than one antibiotic-resistant gene. Our study has notably shown that of all the ESBL genes, the most predominant gene in *E. coli* and *C. freundii* was bla_TEM_, in *Salmonella* spp was a combination of bla_TEM + SHV_, and in *K. pneumoniae*, bla_CTX-M1_ was predominant among the enterobacterial uropathogens isolated from patients of Anambra State University Teaching Hospital, Awka. The genotypic method has a higher specificity/sensitivity than the phenotypic method as thus should be a method of choice for detection of ESBL-producing strains. Limitations of the study are that we didn't record the patient's demographics and history of their antibiotic consumption. We also could not screen specifically for OXA-48 genes.

## Figures and Tables

**Table 1:** Primer sequence/PCR conditions for the ESBL resistance genotyping [12, 40].

Genes	Primer sequences (5′-3′)	Annealing temp. (°C)	No. of cycles (2–4)	Amplicon size (bp)
TEM	F: CATTTCCGTGTCGCCCTTATTC R: CGTTCATCCATAGTTGCCTGAC	60	30	800
SHV	F: AGCCGCTTGAGCAAATTAAAC R: ATCCCGCAGATAAATCACCAC	60	30	713
OXA-1-like	F: GGCACCAGATTCAACTTTCAAG R: GACCCCAAGTTTCCTGTAAGTG	60	30	564
CTX-M-1	F: TTAGGAAATGTGCCGCTGTA R: CGATATCGTTGGTGGTACCAT	60	30	688
CTX-M-2	F: CGTTAACGGCACGATGAC R: CGATATCGTTGGTGGTACCAT	60	30	404
CTX-M-9	F: TCAAGCCTGCCGATCTGGT R: TGATTCTCGCCGCTGAAG	60	30	561
GES 1–9, 11	F: AGTCGGCTAGACCGGAAAG R: TTTGTCCGTGCTCAGGAT	60	30	399
PER 1,3	F: GCTCCGATAATGAAAGCGT R: TTCGGCTTGACTCGGCTGA	60	30	520
VEB 1–6	F: CATTTCCCGATGCAAAGCGT R: CGAAGTTTCTTTGGACTCTG	60	30	648

**Table 2:** Primer sequence/PCR conditions for the MBL, AmpC, KPC, and NDM resistance genotyping [41–42].

Genes	Primer sequences (5′-3′)	Annealing temperature (°C)	No. of cycles (2–4)	Amplicon size (bp)
VIM	F: GAT GGT GTT TGG TCG CAT R: CGA ATG CGC AGC ACC AGA	52	36	390
IMP	F: GGA ATA GAG TGG CTT AAT CTC R: CCA AAC YAC TAS GTT ATC T	52	36	180
GIM	F: TCG ACA CAC CTT GGT CTG AA R: AAC TTC CAA CTT TGC CAT GC	52	36	477
SPM	F: AAA ATC TGG GTA CGC AAA CG R: ACA TTA TCC GCT GGA ACA GG	52	36	271
SIM	F: TAC AAG GGA TTC GGC ATC G R: TAA TGG CCT GTT CCC ATG TG	52	36	570
MOXM	F: GCT GCT CAA GGA GCA CAG GAT R: CAC ATT GAC ATA GGT GTG GTG C	64	25	520
CITM	F: TGG CCA GAA CTG ACA GGC AAA R: TTT CTC CTG AAC GTG GCT GGC	64	25	462
DHAM	F : AAC TTT CAC AGG TGT GCT GGG T R: CCG TAC GCA TAC TGG CTT TGC	64	25	405
ACCM	F: AAC AGC CTC AGC AGC CGG TTA R: TTC GCC GCA ATC ATC CCT AGC	64	25	346
EBCM	F: TCG GTA AAG CCG ATG TTG CGG R: CTT CCA CTG CGG CTG CCA GTT	64	25	302
FOXM	F: AAC ATG GGG TAT CAG GGA GAT G R: CAA AGC GCG TAA CCG GAT TGG	64	25	190
NDM-1	F: ACC GCC TGG ACC GAT GAC CA R: GCC AAA GTT GGG CGC GGT TG	58	35	264
KPC	F: CATTCAAGGGCTTTCTTGCTGC R: ACGACGGCATAGTCATTTGC	55	30	538

**Table 3:** Antibiotic susceptibility pattern of *E. coli* (*n* = 58).

S/no	Antibiotics	No. of isolates (%)		
		Resistant	Intermediate	Susceptible *n*
1	Cefpodoxime (CPD)	35 (60.34)	19 (32.76)	4 (6.90)
2	Ceftriaxone (CTR)	0 (0)	10 (17.24)	48 (82.76)
3	Aztreonam (AT)	1 (1.72)	36 (62.07)	21 (36.21)
4	Cefotaxime (CTX)	8 (13.79)	33 (56.90)	17 (29.31)
5	Ceftazidime (CAZ)	1 (1.72)	34 (58.62)	23 (39.66)
6	Meropenem (MRP)	9 (15.52)	7 (12.06)	42 (72.41)
7	Cefoxitin (CX)	3 (5.17)	11 (18.97)	44 (75.86)
8	Ofloxacin (OF)	4 (6.90)	4 (6.90)	50 (86.21)
9	Ciprofloxacin (CIP)	4 (6.90)	10 (17.24)	44 (75.86)
10	Norfloxacin (NX)	5 (8.62)	1 (1.72)	51 (87.93)
11	Levofloxacin (LE)	4 (6.90)	0 (0)	54 (93.10)
12	Cotrimoxazole (COT)	29 (50)	1 (1.72)	26 (44.83)
13	Gentamicin (GEN)	6 (10.34)	6 (10.34)	45 (77.59)
14	Amoxicillin (AMX)	16 (27.59)	3 (5.17)	20 (34.48)

**Table 4:** Antibiotic susceptibility pattern of *Salmonella* spp. (*n* = 15).

S/no	Antibiotics	No. of isolates (%)		
		Resistant	Intermediate	Susceptible
1	Cefpodoxime (CPD)	0 (0)	0 (0)	15 (100)
2	Ceftriaxione (CTR)	2 (13.33)	0 (0)	13 (86.67)
3	Aztreonam (AT)	0 (0)	2 (13.33)	13 (86.67)
4	Cefotaxime (CTX)	2 (13.33)	0 (0)	13 (86.67)
5	Ceftazidime (CAZ)	1 (6.67)	4 (26.67)	10 (66.67)
6	Meropenem (MRP)	8 (53.33)	1 (6.67)	6 (40)
7	Cefoxitin (CX)	0 (0)	14 (93.33)	1 (6.67)
8	Ofloxacin (OF)	11 (73.33)	4(26.67)	0 (0)
9	Ciprofloxacin (CIP)	8 (53.33)	6 (40)	1 (6.67)
10	Norfloxacin (NX)	8 (53.33)	3 (20)	4 (26.67)
11	Levofloxacin (LE)	9 (60)	2 (13.33)	4 (26.67)
12	Cotrimoxazole (COT)	10 (66.67)	0 (0)	4 (26.67)
13	Gentamicin (GEN)	7 (46.67)	0 (0)	8 (53.33)

**Table 5:** Antibiotic susceptibility pattern of *K. pneumoniae* (*n* = 14).

S/no	Antibiotics	No. of isolates (%)		
		Resistant	Intermediate	Susceptible
1	Cefpodoxime (CPD)	10 (71.43)	0 (0)	0 (0)
2	Ceftriaxione (CTR)	4 (28.57)	4 (28.57)	2 (14.29)
3	Aztreonam (AT)	7 (50)	2 (14.29)	1 (7.14)
4	Cefotaxime (CTX)	9 (64.29)	0 (0)	1 (7.14)
5	Ceftazidime (CAZ)	6 (42.86)	3 (21.43)	1 (7.14)
6	Meropenem (MRP)	4 (28.57)	3 (21.43)	3 (21.43)
7	Cefoxitin (CX)	7 (50)	3 (21.43)	0 (0)
8	Ofloxacin (OF)	5 (35.71)	0 (0)	9 (64.29)
9	Ciprofloxacin (CIP)	5 (35.71)	2 (14.29)	7 (50)
10	Norfloxacin (NX)	5 (35.71)	0 (0)	9 (64.29)
11	Levofloxacin (LE)	5 (35.71)	0 (0)	9 (64.29)
12	Cotrimoxazole (COT)	8 (57.14)	0 (0)	6 (42.86)
13	Gentamicin (GEN)	4 (28.57)	4 (28.57)	5 (35.71)
14	Amoxicillin (AMX)	10 (71.43)	0 (0)	0 (0)

**Table 6:** Summary of multiple antibiotic-resistant indices (MARIs) of uropathogens.

Isolates	Number of isolates (%)	
	MARI > 0.2	MARI ≤ 0.2
*Klebsiella* spp	14 (100)	0 (0)
*E. coli*	33 (57)	25 (43)
*Salmonella* spp	13 (87)	2 (13)
*Citrobacter* spp	7 (70)	3 (30)
*Enterobacter* spp	2 (67)	1 (33)
Total	69 (69)	31 (31)

Total number of antibiotics tested = 14.

**Table 7:** Summary of bla-PCR-positive isolates.

Organisms	TEM	SHV	OXA-1-like	TEM + SHV	TEM + OXA-I-LIKE	TEM + SHV + OXA-1-LIKE	MBL	AmpC
*E. coli* (58)	31	1	0	3	0	0	10	1
*C. freundii* (10)	10	0	0	0	0	0	0	1
*K. pneuminiae* (14)	2	2	0	0	2	3	3	0
*Salmonella* spp (15)	0	0	0	7	0	0	2	0
Total	43	3	0	10	2	3	15	2

**Table 8:** Antibiotic susceptibility pattern of *Citrobacter freundii* (*n* = 10).

S/no	Antibiotics	No. of isolates (%)		
		Resistant	Intermediate	Susceptible
1	Cefpodoxime (CPD)	8 (80)	2 (20)	0 (0)
2	Ceftriaxione (CTR)	4 (40)	4(40)	2 (20)
3	Aztreonam (AT)	3 (30)	6 (60)	1 (10)
4	Cefotaxime (CTX)	6 (60)	2 (20)	2 (20)
5	Ceftazidime (CAZ)	2 (20)	6 (60)	2 (20)
6	Meropenem (MRP)	1 (10)	3 (30)	6 (60)
7	Cefoxitin (CX)	5 (50)	0 (0)	5 (50)
8	Ofloxacin (OF)	0 (0)	3 (30)	6 (60)
9	Ciprofloxacin (CIP)	0 (0)	5 (50)	5 (50)
10	Norfloxacin (NX)	1 (10)	2 (20)	8 (80)
11	Levofloxacin (LE)	0 (0)	1 (10)	9 (90)
12	Cotrimoxazole (COT)	7 (70)	0 (0)	3 (30)
13	Gentamicin (GEN)	1 (10)	0 (0)	9 (90)
14	Amoxicillin (AMX)	8 (80)	0 (0)	2 (20)

**Table 9:** Differences between bla-phenotypic and bla-PCR positives.

Organisms	ESBL		MBL		AmpC	
	Phenotypic positive	PCR-positive	Phenotypic positive	PCR-positive	Phenotypic positive	PCR-positive
*E. coli* (58)	16	35	3	10	2	1
*C. freundii* (10)	5	10	0	0	0	1
*K. pneuminiae* (14)	9	9	0	3	0	0
*Salmonella* spp (15)	1	7	0	2	2	0
Total	31	61	3	15	4	2

## Data Availability

The data used to support the findings of this study are included within the article.
